# Low Autophagy (ATG) Gene Expression Is Associated with an Immature AML Blast Cell Phenotype and Can Be Restored during AML Differentiation Therapy

**DOI:** 10.1155/2018/1482795

**Published:** 2018-03-18

**Authors:** Jing Jin, Adrian Britschgi, Anna M. Schläfli, Magali Humbert, Deborah Shan-Krauer, Jasmin Batliner, Elena A. Federzoni, Marion Ernst, Bruce E. Torbett, Shida Yousefi, Hans-Uwe Simon, Mario P. Tschan

**Affiliations:** ^1^Division of Experimental Pathology, Institute of Pathology, University of Bern, CH-3010 Bern, Switzerland; ^2^Graduate School for Cellular and Biomedical Sciences, University of Bern, CH-3012 Bern, Switzerland; ^3^Department of Molecular and Experimental Medicine, The Scripps Research Institute, La Jolla, CA 92037, USA; ^4^Medi, School for Biomedical Analysts, CH-3014 Bern, Switzerland; ^5^Institute of Pharmacology, University of Bern, CH-3010 Bern, Switzerland; ^6^Members of the Horizon 2020 COST action TRANSAUTOPHAGY (CA15138), Brussel, Belgium

## Abstract

Autophagy is an intracellular degradation system that ensures a dynamic recycling of a variety of building blocks required for self-renewal, homeostasis, and cell survival under stress. We used primary acute myeloid leukemia (AML) samples and human AML cell lines to investigate the regulatory mechanisms of autophagy and its role in AML differentiation. We found a significantly lower expression of key autophagy- (ATG-) related genes in primary AML as compared to healthy granulocytes, an increased autophagic activity during all-*trans* retinoic acid- (ATRA-) induced neutrophil differentiation, and an impaired AML differentiation upon inhibition of ATG3, ATG4D, and ATG5. Supporting the notion of noncanonical autophagy, we found that ATRA-induced autophagy was Beclin1-independent compared to starvation- or arsenic trioxide- (ATO-) induced autophagy. Furthermore, we identified PU.1 as positive transcriptional regulator of ATG3, ATG4D, and ATG5. Low PU.1 expression in AML may account for low ATG gene expression in this disease. Low expression of the autophagy initiator ULK1 in AML can partially be attributed to high expression of the ULK1-targeting microRNA-106a. Our data clearly suggest that granulocytic AML differentiation relies on noncanonical autophagy pathways and that restoring autophagic activity might be beneficial in differentiation therapies.

## 1. Introduction

Basal macroautophagy (thereafter referred to as autophagy), a catabolic recycling system in cells, is key to maintaining cellular homeostasis and survival. Furthermore, activation of autophagy allows to extend cell survival when exposed to different types of stressors such as starvation or cytotoxic drugs. The tightly regulated and dynamic process is characterized by *de novo* formation of autophagosomes. Autophagosomes engulf cytoplasmatic components and deliver these cargos, for example, long-lived proteins or damaged mitochondria, to lysosomes for degradation. Studies in yeast have identified a series of autophagy- (ATG-) related genes forming the autophagy machinery. These ATG genes are highly conserved in mammalian cells, allowing to study their functions also in higher eukaryotes [1–4]. Major steps in the autophagic process include initiation, nucleation, elongation, and maturation of the autophagosomes as well as fusion of the autophagosomes to lysosomes. The process of canonical autophagy follows a hierarchical-ordered recruitment of autophagy-related (ATG) proteins to the phagophore assembly site [5]. The autophagy-initiation complex is composed of ULK1, ATG13, FIP200, and ATG101. The ULK1 protein complex including ULK1, ATG13, and FIP200 coordinates the autophagy initiation from different upstream signaling pathways to induce autophagy [6, 7]. Interestingly, recent data suggest a function for ULK1 not only during autophagy activation but also during elongation and closure of the autophagosomal membrane via binding to ATG8 proteins [8]. Nucleation is under the control of VPS34-Beclin1 class III PI3-kinase complexes resulting in the formation of the isolation membrane [4]. Subsequently, two ubiquitin-like conjugation systems, ATG5/ATG12 and ATG8, the mammalian homologues of which include LC3, GABARAP, and GATE-16, concert the formation of the double-membraned autophagosome [9]. Both systems rely on ATG7, an E1-like enzyme, for activation. Additional proteins involved in these conjugation systems include ATG3, ATG4, ATG10, and ATG16L1. In a last step, autophagosomes fuse with lysosomes to form autolysosomes for the degradation of their contents.

While the importance of autophagy for cell homeostasis and survival has long been appreciated, its role in tumorigenesis and cancer progression is still developing [10, 11]. Autophagy functions in tumor suppression by, for example, preserving protein and organelle homeostasis. Moreover, genome instability was attributed to impaired autophagy and several autophagy genes with tumor suppressor functions (e.g., *BECN1*, *ATG5*, and *ATG4C*) were found [12–14]. On the other hand, cancer cells display increased autophagic activity to meet their increased metabolic needs, and autophagy activated by cytotoxic drugs allows cell survival [15]. Generally, the role of autophagy in tumor development is not fully understood and clearly differs among tumor types and the stage of tumor development. Autophagy may, on the one hand, provide tumor cells with a survival strategy, suggesting a therapeutic use for autophagy inhibition; on the other hand, autophagy may induce cell death by, for example, targeting antiapoptotic proteins, indicating activation of autophagy as novel tool in cancer therapy [16, 17].

Several studies suggested a function for autophagy in mammalian development and cellular differentiation [18–20]. During myelopoiesis, mature myeloid cells undergo a reduction in cell size compared to common myeloid progenitor cells and acquire entirely new morphologies and functions [21, 22]. Such a dramatic change in cell architecture not only implies massive remodeling processes but also requires a delicate balance in macromolecule synthesis and degradation that might be attributed to autophagy. Accordingly, *Atg5* knockout mice showed severe developmental defects [23–25]. In myeloid development, particularly during erythrocyte maturation, the ATG-associated genes *ULK1* (ATG1), *ATG7*, and *NIX* (*BNIPL3*) are critical for the clearance of mitochondria and ribosomes [26, 27]. Furthermore, FIP200, a component of the ULK1 autophagy-initiation complex, is important for hematopoietic stem cell (HSC) maintenance, and *FIP200* knockout HCS displays increased proliferation and myeloid expansion [28]. In general, autophagy is required for HSC survival and the differentiation of adult stem cells including myeloid progenitor cells [6, 20, 27]. Additionally, autophagy is involved in myeloid cell specific functions, such as phagocytosis by monocytes and macrophages [29, 30] as well as antigen presentation by dendritic cells [31]. Lastly, autophagy deficiency led to defects in neutrophil degranulation and reduced the inflammatory potentials of neutrophils [32].

Acute myeloid leukemia (AML) is an aging-related, genetically highly heterogeneous blood cancer subtype that is characterized by the accumulation of myeloid blast cells with altered self-renewal, proliferation, and differentiation function [33]. Acute promyelocytic leukemia (APL), a particular AML subtype, is characterized by the translocation t(15,17) encoding for the oncogene-retinoic acid receptor alpha (PML-RARA) fusion protein [34]. PML-RARA prevents effective transcription of RARA target genes important for myeloid differentiation in a dominant negative manner. Moreover, PML-RARA represses transcription of PU.1 transcriptional targets by binding to overlapping DNA binding sites. Since PU.1 controls transcription of a series of myeloid genes, its inhibition by PML-RARA contributes to the impaired differentiation seen in APL [35]. All-*trans* retinoic acid (ATRA) in combination with anthracyclines or arsenic trioxide (ATO) is able to induce complete remission in 90% of the patients by inducing PML-RARA degradation via the proteasome or caspase cleavage [36, 37]. In addition, ATRA induces Beclin1-independent autophagy or aggrephagy that contributes to the degradation of PML-RARA protein aggregates [38–40]. Furthermore, we and others reported that ATRA-mediated AML differentiation depends on active autophagic flux and that inhibition of autophagy by pharmacological and genetic means attenuated ATRA-induced neutrophil differentiation of APL and non-APL cell lines [40–42]. This also indicates that autophagy is involved in myeloid differentiation beyond its role in the degradation of PML-RARA aggregates [38].

Despite several studies analyzing the function of autophagy during myeloid differentiation [43–48], the myeloid autophagy pathway active during this process is not yet fully characterized, and clinical data on general ATG expression in primary AML are rare. In this study, we show that ATG expression is frequently repressed in primary AML patients and that neutrophil differentiation of AML cells depends on functional autophagy distinct from the canonical pathway. We identified several ATG genes as novel transcriptional targets of PU.1 and speculate that low ATG gene expression in AML is partially due to low PU.1 levels. Lastly, AML differentiation causes downregulation of microRNA-106a, allowing for the expression of its target ULK1. Accordingly, preliminary data indicate that low ULK1 expression is associated with increased miR-106a expression in a small cohort of AML patient samples.

## 2. Results

### 2.1. ATRA-Mediated Cellular Differentiation of AML (HL60) and APL (NB4) Cells Is Associated with Increased, Noncanonical Myeloid Autophagy

**Figure 1:**
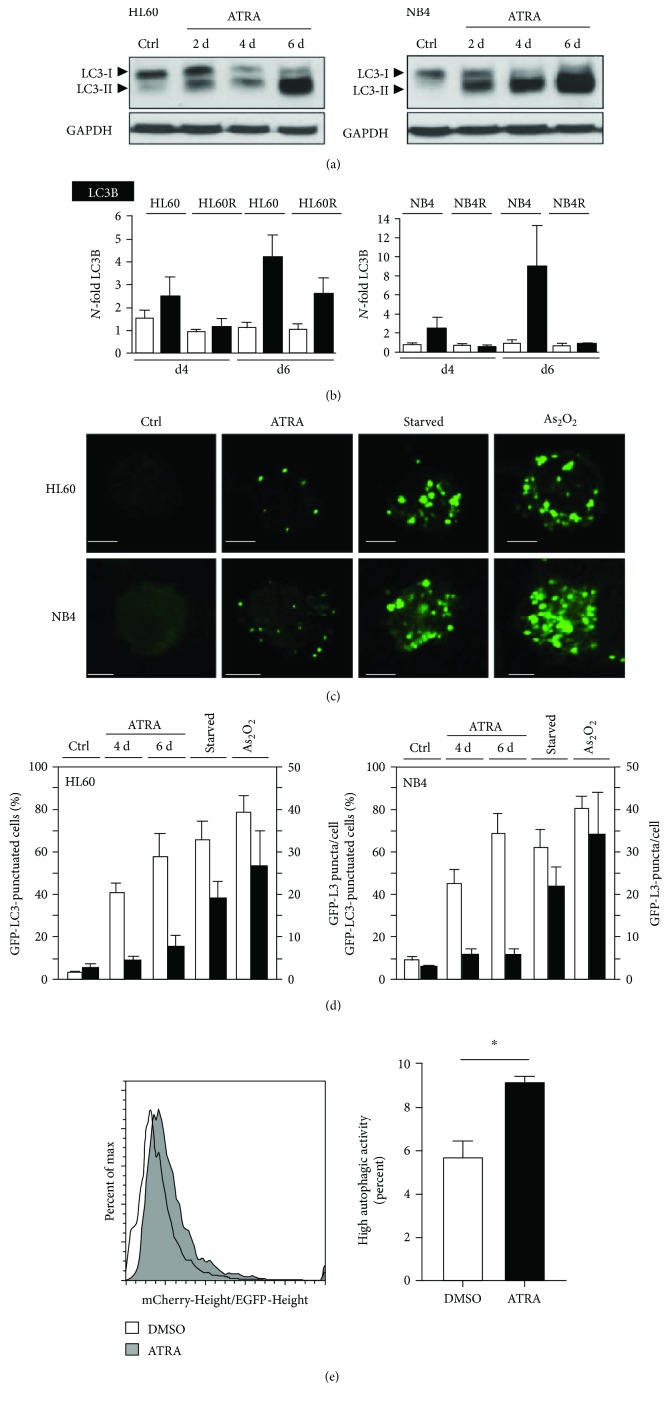
Autophagy is associated with neutrophil differentiation of AML cells. (a) ATRA differentiation induces LC3B lipidation measured by Western blotting. HL60 AML and NB4 APL cells were treated with 1 *μ*M ATRA for up to 6 days. GAPDH was used as a loading control. (b) qPCR analysis of LC3B mRNA of HL60 and NB4 as well as ATRA-resistant HL60-R and NB4-R2 cells after 4 and 6 days of ATRA treatment, respectively. Data are shown as *N*-fold activation compared to untreated cells at days 4 and 6. (c) ATRA differentiation induces GFP-LC3 dot formation. NB4 and HL60 cells stably expressing GFP-LC3 were treated with 1 *μ*M ATRA, starved for 8 hours, or treated with 6 (NB4) and 12 *μ*M (HL60) arsenic trioxide (As_2_O_3_), respectively. GFP-LC3 puncta were detected using confocal microscopy. Scale bar 10 *μ*m. (d) Treatment as in (c). Results are expressed as percentage of cells showing punctuated GFP-LC3 staining and as average number of puncta per cell. Counts are mean ± s.e.m.; *n* = 100; three independent experiments. (e) FACS analysis of NB4 cells expressing the tandem construct mCherry-EGFP-LC3B. Left panel: histogram of the mCherry-Height/EGFP-Height ratio in cells treated with vehicle or with ATRA (1 *μ*M) for 48 h. Right panel: percentages represent cells with high autophagic activity based on a threshold set on control cells. Mann–Whitney *U* test, ^∗^ *p* < 0.05.

To date, activation of autophagic flux during ATRA-induced APL/AML differentiation has mostly been studied during short time exposure to ATRA and was not directly compared to starvation or arsenic trioxide- (ATO-) induced autophagy [38]. Thus, we investigated in more detail how ATRA activates autophagy in non-APL HL60 as well as in APL NB4 cells. We found a marked induction of autophagy during neutrophil differentiation starting from day 2 in both cell lines as indicated by a marked shift from LC3B-I to LC3B-II (Figure 1(a)). As a second autophagy marker, LC3B mRNA induction was analyzed upon ATRA treatment in HL60 and NB4 as well as in their respective ATRA-resistant subclones. LC3B mRNA was significantly upregulated in both parental cell lines upon ATRA treatment but not in the resistant sublines, except for a marked but less pronounced increase at day 6 in the ATRA-resistant HL60 line (Figure 1(b)). Additionally, GFP-LC3B redistributed from a diffuse pattern to a punctuate pattern in HL60 and NB4 cells upon neutrophil differentiation, further indicating active autophagy (Figure 1(c)). Interestingly, quantification of GFP-LC3 dots revealed that starvation- and ATO-induced autophagy in the same cells was clearly different from ATRA-mediated induction of autophagy (Figure 1(d), black bars). Still, the percentage of cells with GFP-LC3B puncta was similar for all three treatments (Figure 1(d), white bars). Lastly, to confirm induction of autophagy flux upon ATRA treatment, we generated NB4 cells expressing the mCherry-EGFP-LC3B tandem construct. Autolysosomes appear red since the lower pH in these organelles quenches the EGFP signal. The ratio of mCherry to EGFP fluorescence was determined by flow cytometry as described earlier [49]. A threshold was set to identify the percentage of cells with high autophagic activity (high mCherry/EGFP ratio). We found a shift towards red fluorescence in NB4 cells treated with ATRA (Figure 1(e), left panel) together with a significant increase of cells with high autophagic activity (Figure 1(e), right panel).

To test whether autophagy is essential for neutrophil differentiation of HL60 AML cells, we blocked autophagy pharmacologically using the phosphatidylinositol 3-kinase (PI3K) inhibitor 3-methyladenine (3-MA) or chloroquine (CQ). Inhibition of ATRA-induced autophagy resulted in diminished neutrophil differentiation of these cells as evidenced by significantly reduced CD11b surface expression (Supplementary Figure 1a). In order to exclude that inhibiting autophagy influenced neutrophil differentiation solely by increasing apoptosis, we cotreated these cells with the pan-caspase inhibitor z-VAD-fmk. Blocking apoptosis during 3-MA or CQ-mediated inhibition of ATRA-induced autophagy did not show any effects on neutrophil differentiation (Supplementary Figures 1a and b, and data not shown). Our data clearly suggest that induction of autophagy is a prerequisite for ATRA-induced neutrophil differentiation of AML cells per se whereas its role in ATRA-induced cell death is negligible.

### 2.2. ATG Gene Expression Is Frequently Reduced in Primary AML Patients

**Figure 2:**
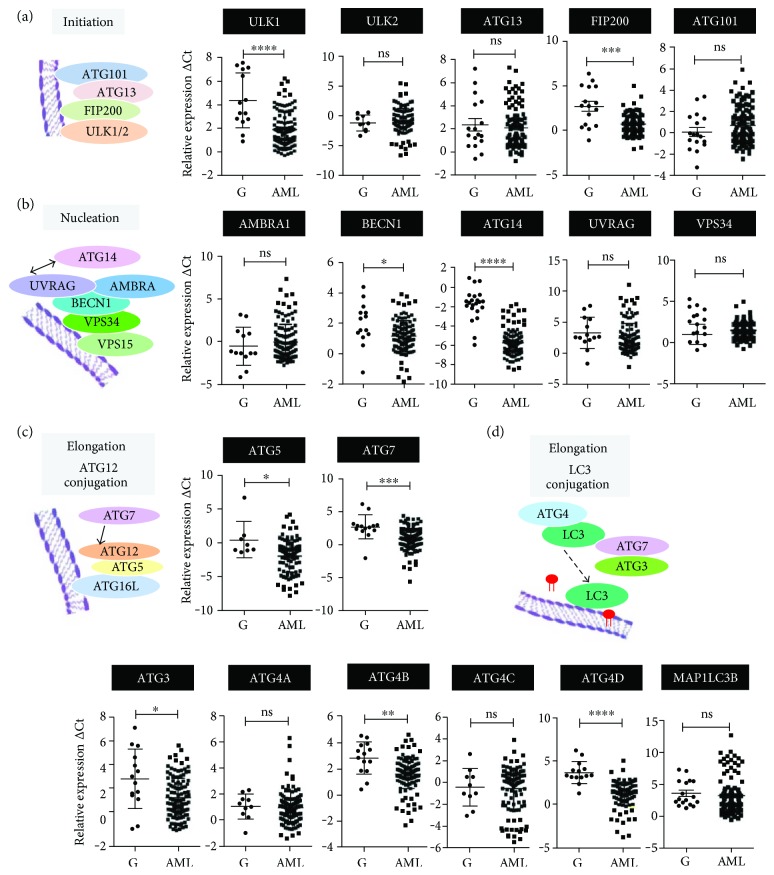
Autophagy- (ATG-) related gene expression is frequently inhibited in clinical AML samples. Primary AML blasts were isolated using a Ficoll gradient; total RNA was extracted; and ATG gene mRNA levels during autophagy initiation (a), nucleation (b), ATG12- (c), and LC3 (d) conjugation phases were quantified by qPCR. Measured cycle threshold (Ct) values represent log2 expression levels. Values were normalized to the expression levels of the housekeeping genes HMBS and ABL1. Mann–Whitney *U* test, ^∗^ *p* < 0.05, ^∗∗^ *p* < 0.01, ^∗∗∗^ *p* < 0.001, ^∗∗∗∗^ *p* < 0.0001.

We previously published that ATG genes such as *DRAM1*, *WIPI1/2*, and *MAP1S* were significantly downregulated in primary AML samples [42, 50, 51]. Nevertheless, a more global ATG gene expression profile in clinical AML patient samples is still missing. Therefore, we quantified the expression levels of additional 18 ATG genes in a cohort of 114 AML patient samples with defined chromosomal aberrations and compared them to the corresponding expression levels in mature granulocytes from healthy donors (*n* = 13) (Figures 2(a)–2(d) and Supplementary Figure 2). The expression of 9/18 ATG genes, operative in different phases of autophagosome biosynthesis, was significantly inhibited in AML as compared to their expression in mature, healthy granulocytes: *ULK1, FIP200, BECN1, ATG14, ATG5, ATG7, ATG3, ATG4B, and ATG4D*. Our data suggest that low ATG gene expression is associated with an immature AML blast phenotype.

### 2.3. Granulocytic Differentiation of AML/APL Cells, Primary Human APL Cells, and Healthy Human CD34^+^ Progenitor Cells Is Paralleled by Increased ATG Gene Expression

**Figure 3:**
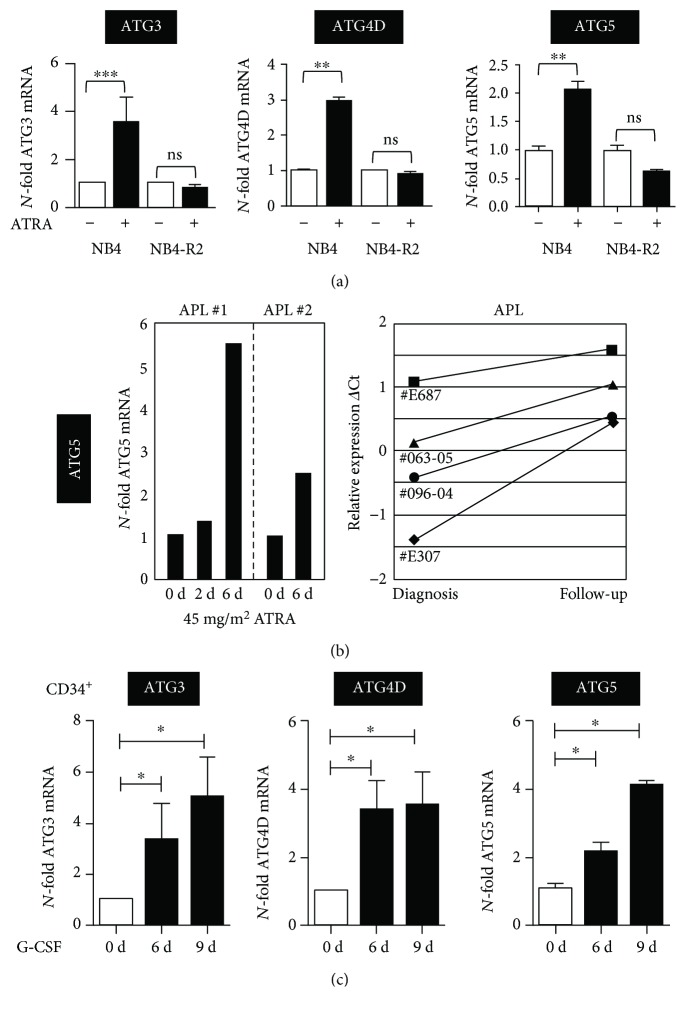
Induction of *ATG3*, *ATG4D*, and *ATG5* gene expressions upon neutrophil differentiation of normal and leukemic precursor cells. (a) *ATG3*, *ATG4D*, and *ATG5* qPCR analyses of NB4 and ATRA-resistant NB4-R2 cells upon treatment with 1 *μ*M ATRA for 4 days. Results are given as *N*-fold changes compared with untreated SHC002 control cells and normalized to the housekeeping gene *HMBS*. (b) Induction of *ATG5* during ATRA therapy of APL patients. Two patients with newly diagnosed APL t(15;17) were treated with orally administered tretinoin (ATRA) at a dosage of 45 mg/m^2^ daily. Total RNA was extracted from blast cells isolated using a Ficoll gradient, and expression levels of *ATG5* and were assessed by qPCR. Values were normalized to *HMBS* and day 0 as the experimental starting point (left panel). A similar induction of *ATG5* mRNA was seen in 4 APL patients at diagnosis and after finishing ATRA therapy (mean 1.3 months; right panel). Relative expression is given as differences in Ct values between *ATG5* mRNA and the levels of the housekeeping genes HMBS and ABL1 (ΔCt) representing log2 expression levels. (c) Normal CD34^+^ progenitor cells were differentiated with G-CSF for indicated days; and *ATG3*, *ATG4D*, and *ATG5* mRNA expressions were determined by qPCR. Data analysis as in (a). Mann–Whitney *U* test, ^∗^ *p* < 0.05, ^∗∗^ *p* < 0.01, ^∗∗∗^ *p* < 0.001.

Based on our findings in clinical AML samples showing globally reduced ATG gene transcription compared to mature granulocytes, we asked if these genes are induced during leukemic and normal neutrophil differentiation. To this end, we first analyzed expression of selected ATG genes, ATG3, ATG4D, and ATG5 during neutrophil differentiation in APL cell lines as well as in primary APL patients receiving ATRA therapy. Due to limited patient sample RNA, we could only determine *ATG5* expression in APL patients. We found that all three ATG genes investigated were significantly induced upon 4 days of ATRA treatment in parental but not in ATRA-resistant NB4 cells (NB4-R2) (Figure 3(a)). Importantly, marked *ATG5* induction was seen in primary APL patients upon ATRA therapy *in vivo* during short- and long-term follow-up examinations (Figure 3(b)). Lastly, to test if ATG gene expression is also induced during normal granulocytic differentiation, we differentiated human CD34^+^ progenitor cells towards neutrophils using G-CSF. Similar to leukemic neutrophil differentiation, all three ATG genes analyzed showed a significant increase in their expression levels (Figure 3(c)). Together, neutrophil differentiation is positively correlated with increased expression of key ATG genes.

### 2.4. Inhibiting the Key ATG Gene ATG5 Significantly Attenuates Granulocytic Differentiation and Autophagy of AML Cells

**Figure 4:**
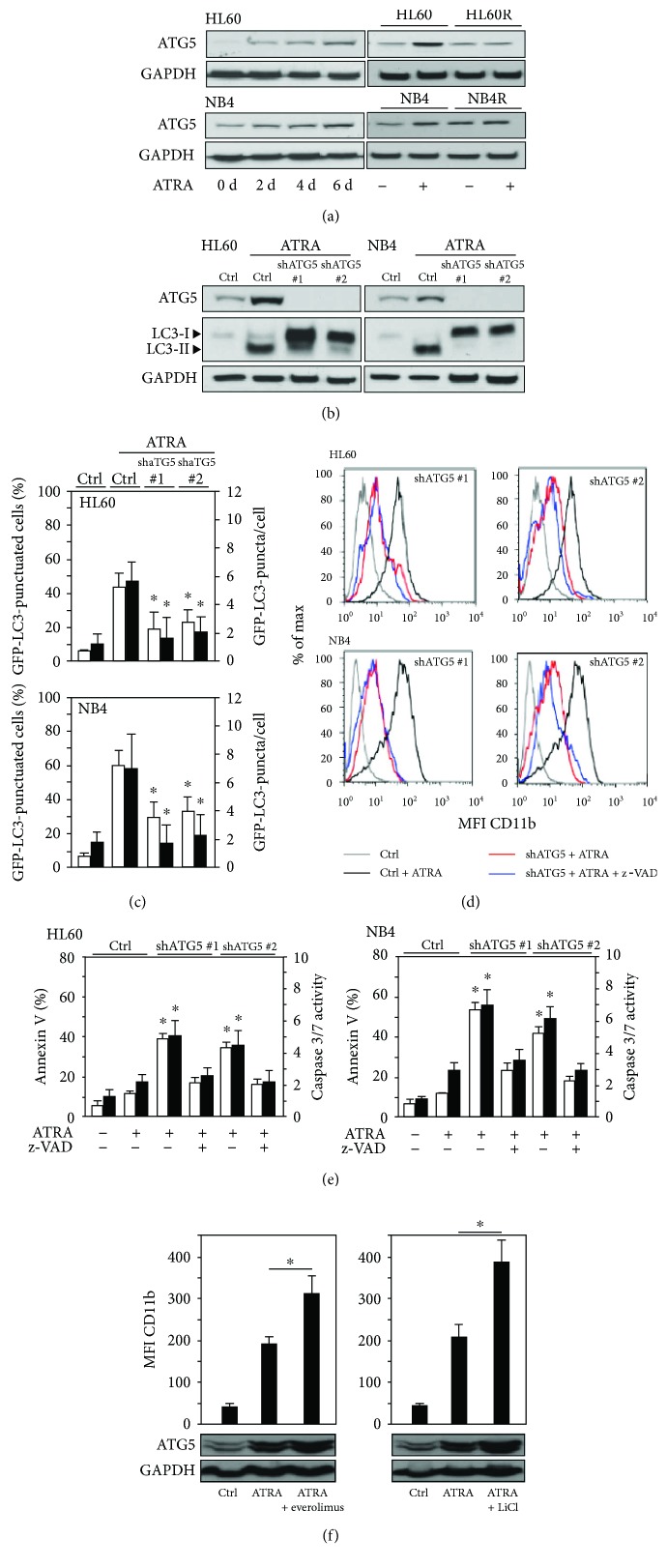
ATG5 induction is essential for neutrophil differentiation AML cells. (a) ATG5 protein expression in HL60, NB4, and the ATRA-resistant NB4-R2 and HL60-R upon ATRA-induced neutrophil differentiation. Cells were treated with 1 *μ*M ATRA for up to 6 days. At time points indicated, proteins were extracted and ATG5 levels were analyzed by Western blotting. GAPDH was used as a loading control. (b) Inhibition of ATG5 precludes ATRA-induced autophagy. Cells stably expressing a scramble control shRNA (Ctrl) or shRNAs targeting *ATG5* (shATG5_1 and shATG5_2) were treated with ATRA for 4 days. Western blotting for ATG5 and LC3B is shown. GAPDH was used as a loading control. (c) Inhibition of ATG5 prevents ATRA-induced autophagy as measured by GFP-LC3 dot formation. NB4 and HL60 GFP-LC3 cells expressing scramble control shRNA (shCtrl) or shRNAs targeting *ATG5* (shATG5_1 and shATG5_2) were treated as in (b). The percentage of GFP-LC3 puncta-positive cells and average numbers of puncta were quantified by confocal microscopy. Counts are mean ± s.e.m.; *n* = 100; three independent experiments. (d) Inhibition of ATG5 impairs neutrophil differentiation of AML cells. FACS analysis of CD11b expression in cells treated as in (b) is shown. Blocking apoptosis by z-VAD-fmk did not alter reduced differentiation in ATG5 knockdown cells. Data are mean ± s.e.m.; *n* = 1 × 10^4^. (e) HL60 and NB4 *ATG5* knockdown AML cells displayed increased apoptosis upon ATRA treatment. Apoptosis was determined by annexin V staining and caspase 3/7 activity. z-VAD-fmk treatment efficiently attenuated apoptosis induction in *ATG5* knockdown cells during ATRA-induced differentiation. Cells treated as in (b). (f) Pharmacological activation of autophagy enhances neutrophil differentiation of AML cells. HL60 cells were treated with 1 *μ*M ATRA alone or in combination with 0.5 *μ*M everolimus (left panel) or 25 mM lithium chloride (right panel) for 4 days. The combination treatment significantly enhanced neutrophil differentiation as measured by CD11b induction. ATG5 induction was assessed by Western blotting. Data are mean ± s.e.m. of three independent experiments. Mann–Whitney *U* test, ^∗^ *p* < 0.05; n.s.: not significant.

To test if ATRA-induced autophagy depends on the conserved elongation process during autophagy, we knocked down *ATG5* in two AML differentiation models. We first analyzed ATG5 protein expression during neutrophil differentiation of HL60 AML and NB4 APL cells. We found a time-dependent upregulation of ATG5 protein levels in both cell lines during ATRA differentiation (Figure 4(a), left panels). To exclude that ATG5 induction is solely a consequence of a stress response to ATRA treatment rather than being functional in myeloid differentiation, we analyzed ATG5 expression in ATRA-resistant HL60-R and NB4-R2 sublines. Both cell lines did not upregulate ATG5 upon ATRA treatment compared to the parental cells (Figure 4(a), right panels), further suggesting that upregulation of ATG5 is associated with neutrophil differentiation. Importantly, knocking down *ATG5* using two independent shRNAs significantly reduced ATRA-induced autophagy (Figures 4(b) and 4(c)). In parallel with disabling ATRA-induced autophagy, AML *ATG5* knockdown cells showed impaired neutrophil differentiation (Figure 4(d)). Moreover, inhibition of apoptosis using caspase inhibitors had no effect on reduced neutrophil differentiation upon ATG5 inhibition (Figures 4(d) and 4(e)), suggesting that reduced cellular differentiation is not due to increased cell death of *ATG5* knockdown cells. Importantly, combining ATRA with the pharmacological autophagy inducers everolimus or LiCl resulted in significantly enhanced neutrophil differentiation as assessed by elevated CD11b surface expression paralleled by increased ATG5-ATG12 complex formation (Figure 4(f)).

To further validate earlier findings regarding the type of autophagy activated by ATRA, we knocked down *Beclin1* in HL60 AML and NB4 APL cells. As described above, we found significantly lower *Beclin1* levels in primary AML samples as compared to mature neutrophils. In line with these data, we observed slightly increased Beclin1 protein upon ATRA-induced neutrophil differentiation of both AML cell lines (Supplementary Figure 3a). Importantly, knocking down *Beclin1* did not abrogate ATRA-induced autophagy (Supplementary Figures 3b and c, left panels). At the same time, inhibition of Beclin1 significantly reduced starvation- and arsenic trioxide- (ATO-) induced autophagy in HL60 and NB4 cells, confirming the functionality of the *Beclin1* knockdown (Supplementary Figure 3c, right panels). In line with its negligible role in ATRA-induced autophagy, *Beclin1* knockdown AML cells displayed no significant reduction in neutrophil differentiation (Supplementary Figure 3d). Thus, ATRA-induced autophagy seems clearly different—less intense and Beclin1-independent—from canonical starvation- or ATO-induced autophagy.

### 2.5. Transcriptional Regulation of Key ATG Genes by the Myeloid Master Regulator PU.1

**Figure 5:**
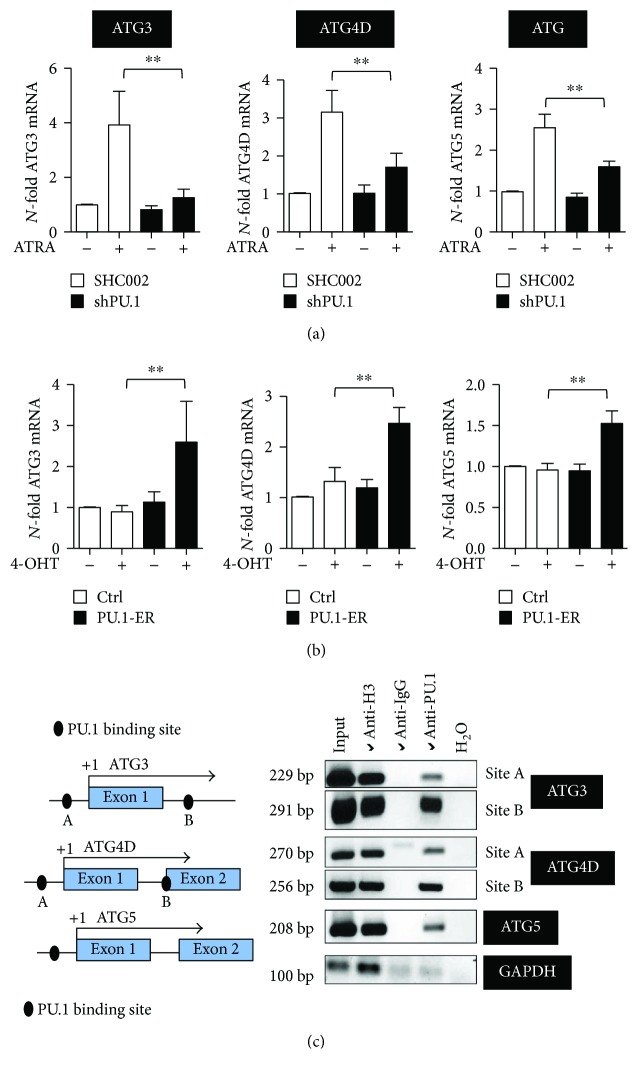
PU.1-dependent regulation of *ATG3*, *ATG4D*, and *ATG5* during ATRA-mediated differentiation of NB4 cells. (a) *ATG3*, *ATG4D*, and *ATG5* mRNA expression levels were quantified in NB4 shPU.1 cells treated with ATRA for 4 days. (b) NB4 cells, transduced with an inducible PU-1-ER expressing vector, were treated with 4-OHT to induce PU.1 translocation to the nucleus. *ATG3*, *ATG4D*, and *ATG5* mRNA expression levels were quantified as in (a). (c) Schematic representation of *ATG3*, *ATG4D*, and *ATG5* proximal promoter regions. Putative PU.1 binding sites in these promoter regions are indicated as black circles. *In vivo* binding of PU.1 to the indicated PU.1 binding sites was shown by ChIP in NB4 cells using antibodies against PU.1. Antibodies against acetyl-histone H3 and IgG are used as positive and negative controls, respectively. GAPDH amplification was shown as a negative control for the different pull-downs. Mann–Whitney *U* test, ^∗∗^ *p* < 0.01.

The Ets family transcription factor PU.1, a master regulator of myeloid cell development, is significantly downregulated in AML. Our earlier findings that the autophagy-associated genes *MAP1S* and *WIPI1* are PU.1 transcriptional targets as well as our findings that knocking down *PU.1* significantly attenuates ATRA-mediated autophagic flux [42] prompted us to further investigate if additional ATG genes are regulated by this transcription factor. First, we determined ATG3, ATG4D, and ATG5 gene expressions in two independent NB4 *PU.1* knockdown cell lines upon ATRA treatment. Induction of all three genes was significantly reduced at the mRNA level when *PU.1* was knocked down (Figure 5(a)). To verify PU.1-dependent expression of these ATG genes, we used NB4 cells expressing an inducible PU.1-ER construct, which can be activated upon tamoxifen treatment leading to PU.1-ER translocation to nucleus and transcriptional activity. mRNA levels of all three genes were significantly induced upon PU.1-ER activation in NB4 cells (Figure 5(b)). We then analyzed a 3.5 kb genomic region up- and downstream of the transcriptional start site of all three ATG genes using MatInspector. We identified several putative PU.1 binding sites (Figure 5(c), left panel). PU.1 chromatin immunoprecipitation revealed binding of PU.1 to the promoter region of *ATG3*, *ATG4D*, and *ATG5* (Figure 5(c)). In summary, we identified three additional ATG genes that are regulated by PU.1 during ATRA-induced neutrophil differentiation of AML cells.

### 2.6. Posttranscriptional Regulation of ULK1 by miR-106a during AML Differentiation

**Figure 6:**
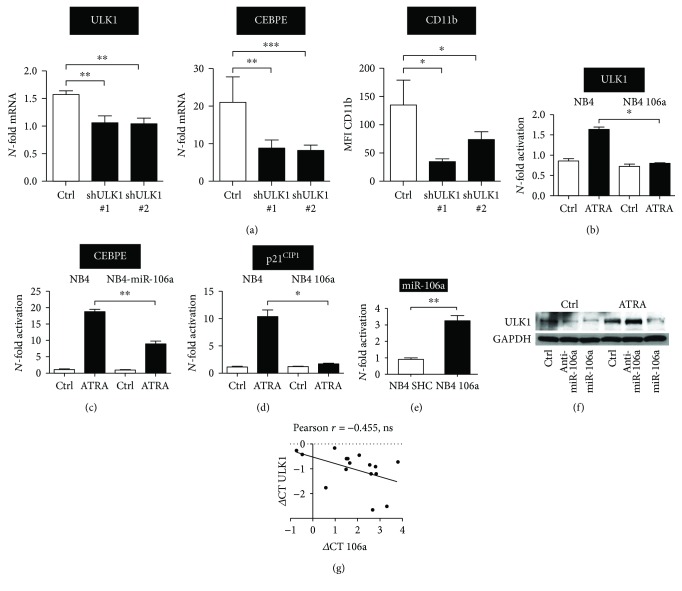
miR-106a targets *ULK1* and attenuates ATRA-induced AML differentiation. (a) Inhibition of ULK1 attenuates neutrophil differentiation of APL cells. NB4 cells stably expressing a scramble control shRNA (Ctrl) or shRNAs targeting *ULK1* (shULK1 #1 and shULK1 #2) were treated with ATRA for 4 days. qPCR for *ULK1* and *CEBPE* as well as FACS analysis of CD11b is shown. Data are mean ± s.e.m. of three independent experiments. (b) *ULK1*, *CEBPE*, and *p21^CIP1^* expressions were detected by qPCR in NB4 cells stably transduced with a scrambled control (NB4) or a lentiviral vector expressing miR-106a precursors (NB4 106a). Cells were treated with 1 *μ*M ATRA for 4 days. Results were normalized to *HMBS* and are shown as *N*-fold change relative to the corresponding untreated control cell line. (e) qPCR analysis of miR-106a expression in NB4 control and miR-106a overexpressing NB4 cells. (f) Western blot analysis of ULK1 expression in control, anti-miR-106a, and miR-106a-expressing NB4 cells upon ATRA treatment for 4 days. GAPDH was used as a loading control. (g) Total RNA was isolated from 16 AML patients, and qPCR was performed to determine miR-106a and *ULK1* mRNA levels, respectively. SNORA38B and 5s rRNA were used as reference RNAs for the microRNA qPCR and *HMBS* and *ABL1* as reference mRNAs for the mRNA qPCR. ΔCt values of *ULK1* and miR-106a were calculated and plotted against each other. Linear regression and Pearson *r* were calculated using Prism software. Mann–Whitney *U* test, ^∗^ *p* < 0.05, ^∗∗^ *p* < 0.01, ^∗∗∗^ *p* < 0.001.

Given the widespread aberrant microRNA (miRNA) expression in myeloid malignancies, we asked if altered miRNA expression in AML might contribute to ATG gene repression in this disease. Based on a miRNA profiling study that identified the miR-17 and miR-181 family members as the most downregulated miRNAs during neutrophil differentiation of NB4 APL cells [52], and our study identifying *ULK1* as a novel target of the miR-17 family member miR-106a in lung cancer therapy [53], we hypothesized that this miRNA also targets *ULK1* in AML. In a first step, we evaluated if knocking down *ULK1* similar to the other ATG genes analyzed would attenuate neutrophil differentiation. Indeed, using two independent shRNAs targeting ULK1, we found that inhibiting ULK1 resulted in significantly reduced *CEBPE* and CD11b levels (Figure 6(a)). Moreover, miR-106a expression prevented ULK1 induction upon ATRA-induced differentiation of NB4 paralleled by impaired neutrophil differentiation as seen by significantly reduced induction of *CEBPE* mRNA compared to control transduced cells (Figures 6(b) and 6(c)). The expression of the known miR-106a target p21^CIP1^, a gene induced during neutrophil differentiation, was determined as a positive control for the functionality of the miR-106a vector used (Figure 6(d)). Ectopic expression of miR-106a in NB4 cells is shown in Figure 6(e). Accordingly, using an anti-miR-106a construct, we found that blocking miR-106a resulted in increased protein expression of ULK1 during ATRA treatment whereas overexpression of miR-106a resulted in markedly reduced ULK1 expression (Figure 6(f)). Lastly, our preliminary data indicate that *ULK1* mRNA is negatively associated with miR-106a expression in a cohort of 16 primary AML patient samples (Figure 6(g)). Our studies clearly indicate that ULK1 is functional in neutrophil differentiation of AML cells and that it is targeted by the oncogenic miR-106a, providing a possible explanation for low ULK1 expression levels in AML.

## 3. Discussion

In this study, we identified a Beclin1-independent, low-intensity autophagy during neutrophil differentiation of AML cells. We determined the expression of key ATG genes involved in different autophagy phases in a large panel of primary AML patients. Overall, we found that the expression of a variety of ATG genes is significantly lower in primary AML as compared to normal granulocytes. The expression of these ATG genes is restored in AML cells upon induction of neutrophil differentiation paralleled by an activation of the autophagic flux. Moreover, we found that the Ets family transcription factor PU.1, a master regulator of myeloid differentiation, positively regulates expression of several ATGs. These findings add ATGs to the list of PU.1-regulated genes that are important for neutrophil differentiation. Thus, decreased ATG gene expression can partially be attributed to low expression of PU.1 in AML patients. We also showed that miR-106a targets the important autophagy gene ULK1 in AML. Together, we provide first explanations for low ATG expression in AML cells.

The role of autophagy during cell differentiation and mammalian development [18] has been long appreciated, and several studies show that disruption in autophagy function contributes to a cellular differentiation block. For example, autophagy is needed for elimination of mitochondria in red cell precursors, and deficiency in autophagy genes impairs erythrocyte maturation and causes anemia in mice [54]. During megakaryopoiesis and thrombopoiesis, autophagy is required for proper cell cycle and mitochondrial function, and a study using Atg7-deficient mice shows that autophagy deficiency causes impaired platelet production and function [43]. Accordingly, megakaryocytic differentiation of the chronic myelogenous leukemia cell line K562 needs increased autophagy function [55]. During monocyte-macrophage differentiation, autophagy is induced upon release of Beclin1 from Bcl-2 and promotes not only cellular differentiation but also cell survival [45]. Another study using conditional *Atg5* knockout in myeloid progenitors described a mild expansion of precursor cells [56]. We and others showed that autophagy is crucial for therapy-induced neutrophil differentiation of AML cells indicating a similar dependence on autophagy for neutrophil development as in healthy individuals [38–40, 42]. Whether there are different functions for ATG5 or noncanonical autophagy in general during normal versus leukemic neutrophil differentiation is still under investigation.

The phosphoinositide-3-kinases (PI3K)/AKT and mTOR signaling pathways are currently targeted in clinical trials to treat AML by increasing cell death in combination with chemotherapy [57]. Since our results showed decreased ATRA-mediated differentiation when autophagy is inhibited, autophagy modulation in cancer therapy could have a potential negative effect on neutrophil differentiation. In general, class I PI3K inhibits autophagic initiation, whereas the class III enzymes stimulate autophagic activity. The net effect of broad-spectrum PI3K inhibitors targeting both classes of PI3K typically induces a block in autophagy. This block in autophagy might interfere with differentiation therapy. Nevertheless, specific inhibitors of class I PI3K had no impact on ATRA differentiation of APL cells and might be considered to enhance cell death in combination with chemotherapy [58]. Concerning our findings, we are confident that reduced ATRA-induced autophagy and neutrophil differentiation upon cotreatment with ATRA and 3-MA/wortmannin resulted in the inhibition of class III enzymes leading to a block of autophagy initiation. Our hypothesis is supported by earlier findings showing that knocking down *PIK3C3* resulted in a similar differentiation block as seen with 3-MA treatment [42]. Why does inhibition of *PIK3C3* but not of Beclin1 attenuate neutrophil differentiation despite that both proteins are part of the autophagy-initiation complex? Beclin1 might not be the limiting factor for the PIK3C3 nucleation complex in ATRA-induced autophagy. In favor of this explanation, we observed an increase in ATG5-ATG12-conjugated protein upon inhibition of Beclin1 (data not shown), suggesting that cells compensate the loss of Beclin1 by upregulation of other autophagy-related proteins. Similarly, small amounts of Beclin1 protein, which are released from Bcl-2 inhibition through ATRA-mediated downregulation of Bcl-2 [59], could be sufficient to activate and stabilize the PIK3C3 complex. Further, *PIK3C3* not only is involved in autophagosome nucleation but also acts at several steps along the signaling pathway associated with autophagy [60]. Thus, it seems possible that the *PIK3C3* knockdown phenotype during neutrophil differentiation represents the consequences of blocking autophagy at later stages.

Our findings in HL60 non-APL cells suggest that AML differentiation requires functional autophagy not only for the degradation of PML-RARA found in APL. Blocking autophagy interferes with differentiation, for example, with cell cycle arrest (data not shown), and results in cell death. Autophagy may protect malignant HSCs with a normal autophagy machinery or cancer cells under stress. However, autophagy is also important for maintenance of normal HSCs [61, 62]. Inhibition of autophagy by Bafilomycin A1 had no impact on differentiation, and VitD3 triggered autophagy resulting in Beclin1-dependent cell death. In line with previous finding that inhibition of autophagy results in apoptosis of cells that are engaged in differentiation [63], we now show a beneficial effect of cotreatment with ATRA and autophagy inducers. This differentiation-enhancing effect by combined activation of differentiation and autophagy might have clinical implications. A considerable number of APL patients present with major complications during differentiation therapy (e.g., ATRA syndrome or APL differentiation syndrome, 10–15%) [64]; thus reducing ATRA concentration in combination with autophagy activators might be beneficial for these patients. Generally, enhancing autophagy with FDA-approved drugs may represent a possible new strategy to treat APL patients and possibly sensitize additional AML subtypes to ATRA treatment. Promising autophagy-enhancing compounds to use in combination with ATRA include rapalogs (sirolimus, temsirolimus, and everolimus) and Ca^2+^ channel blockers (verapamil, loperamide, and pimozide) [65].

In conclusion, our studies show that increased ATG gene expression is associated with normal neutrophil differentiation, and that differentiation of AML cells involves a noncanonical autophagy pathway, which is Beclin1-independent. Increasing levels of ATG3, ATG4D, and ATG5 mRNA during neutrophil differentiation of healthy CD34^+^ hematopoietic progenitor cells and their high expression in mature neutrophils point to a more general role for autophagy not only during leukemic but also during normal neutrophil differentiation. Key autophagy genes such as *ULK1*, *ATG3*, *ATG4D*, or *ATG5* are significantly downregulated in primary AML patient samples, and their expressions can be restored upon ATRA therapy in APL patients and AML cell lines. The low expression of these genes is partially due to inhibition of their positive regulator PU.1, or in the case of ULK1, by increased expression of its negative regulator miR-106a. Clearly, ATRA-induced myeloid autophagy is different from starvation- or chemotherapeutic-induced autophagy, and further investigations are needed to clarify its functions and to elucidate the signaling pathways involved. This is an important task since knowing the exact noncanonical autophagy pathway will serve to develop more specific and improved autophagy drugs. Finally, clinical applications planning to inhibit autophagy in order to decrease cell survival may need to consider detrimental effects on myeloid differentiation.

## 4. Materials and Methods

### 4.1. Primary Patient Samples, CD34^+^ Cells, and Cell Lines

Primary AML patient cDNA samples were obtained from a cohort of AML patient samples from HOVON/SAKK (Dutch-Belgian Hematology-Oncology/Swiss Group for Clinical Cancer Research Cooperative group) protocols 04, 04A, 29, and 42 (available at http://www.hovon.nl) between 1987 and 2006. All patients provided written informed consent in accordance with the Declaration of Helsinki. Patient data represent log2 expression levels and were normalized to the expression levels of the 2 housekeeping genes *HMBS* and *ABL*. CD34^+^-mobilized peripheral blood cells from healthy donors were expanded, cultured, and differentiated. The human APL cell lines NB4 and its ATRA-resistant clone NB4-R2, and the AML M2 cell line HL-60 and its ATRA-resistant clone HL60-R were maintained in RPMI-1640 (Sigma-Aldrich), supplemented with 10% fetal bovine serum (FBS), 50 U/ml penicillin, and 50 *μ*g/ml streptomycin (Sigma-Aldrich), in a humidified incubator containing 5% CO_2_ at 37°C. The human embryonic kidney 293T cells were cultured in DMEM (Sigma-Aldrich) supplemented with 5% FBS, 1% penicillin/streptomycin, and 1% HEPES (Sigma-Aldrich) and kept in a humidified atmosphere containing 7.5% CO_2_ at 37°C.

For neutrophil differentiation, AML parental and knockdown cell lines were seeded at a density of 0.2 × 10^6^/ml and treated with 1 *μ*M ATRA (Sigma-Aldrich) for 2–6 days as indicated. Neutrophil differentiation was assessed by increased CCAAT/enhancer binding protein epsilon (CEBPE) mRNA expression and by CD11b FACS analysis. Arsenic trioxide (As_2_O_3_; Sigma) was dissolved in 1 M NaOH and used at 6–12 *μ*M. 3-Methyladenine (3-MA; Sigma) was dissolved in H_2_O and used at 5 mM. Chloroquine diphosphate salt (CQ; Sigma) was dissolved in H_2_O and used at 25 *μ*M. Bafilomycin A1 was dissolved in DMSO, used at 200 nM, and added 2 hours before analysis.

### 4.2. Lentiviral Vectors, Lentivirus Preparation, and Transduction of Cell Lines

pLKO.1 lentiviral vectors expressing small hairpin (sh) RNAs targeting PU.1, ATG5, or Beclin1 and a nontargeting shRNA control (SHC002) were purchased from Sigma-Aldrich. These vectors contain a puromycin antibiotic-resistant gene for selection of transduced mammalian cells. PU.1-ER construct was generated by subcloning PU.1-ER fragment into pLV-EF1a-MCS-IRES-Hyg vector containing hygromycin antibiotic-resistant gene using the In-Fusion HD cloning kit (Takara) according to the manufacturer's instruction. Lentivirus production and transduction were done as described [66]. Transduced AML cell line populations were selected with 1.5 *μ*g/ml puromycin for 4 days or with 250 *μ*g/ml hygromycin for 10 days. Knockdown efficiency was assessed by Western blot and/or qPCR analysis. An mCherry-EGFP-LC3-expressing lentiviral vector was kindly provided by Dr. Maria S. Soengas (CNIO, Molecular Pathology Program, Madrid, Spain).

### 4.3. RNA Extraction and Quantitative RT-PCR (qPCR)

Total mRNA was isolated using miRCURY™ RNA isolation kits (Exiqon) according to the manufacturer's instruction. Total RNA was reverse transcribed, and ATG gene expression in AML patients was quantified using RT-PCR low-density arrays to quantify ATG gene expression as described previously [67]. For quantification of *ATG3*, *ATG4D*, *ATG5*, and *CEBPE* expression in cell line experiments, the TaqMan® Gene Expression Assays Hs00223937_m1, Hs00262792_m1, Hs00169468_m1, and Hs00357657_m1 were used, respectively. Specific primers and probes for *HMBS* and *PU.1* have been described [66]. miRNA expression was assessed using the miScript SYBR Green PCR kit and primer assay hsa-miR-106a (Qiagen). We used hsa-miR-SNORA-73A as a housekeeping gene for miRNA normalization. *N*-fold changes were calculated using the ΔΔCt method of relative quantification. Data represent the mean ± s.e.m. of at least triplicate experiments.

### 4.4. Western Blot Analysis

Whole cell extracts were washed in ice-cold PBS and lysed using urea lysis buffer consisting of 8 M urea and 0.5% Triton X-100 supplemented with protein inhibitor cocktail (Roche Diagnostics). The lysates were sonicated for 3 s and then centrifuged at 13,000 rpm for 15 minutes at 4°C. Bradford assay with BSA as a standard was used to determine the concentration of protein contents. 40 *μ*g of total proteins was analyzed by electrophoresis on precast gel (Biorad). Blots were incubated with the primary antibody anti-LC3B (NB600-1384; Novus Biologicals) in TBS 0.05 Tween-20/5% milk or with anti-PU.1 in TBS 0.05 Tween-20/3% BSA overnight at 4°C and then incubated with secondary antirabbit DyLight 650 for 1 hour at room temperature. Additional antibodies used were anti-Beclin1 (Cell Signaling, #3738), anti-ATG5 (Sigma, A2856), and mouse monoclonal anti-GAPDH (Sigma). Blots were imaged using the ChemiDoc (Biorad) and Image Lab software.

### 4.5. Immunocytochemistry, Confocal Microscopy, and Image Analysis

We followed the methods of Wampfler et al. [68]. Briefly, NB4 cells were fixed in 2% paraformaldehyde in PBS and permeabilized in 0.1% Triton X in PBS or fixed and permeabilized in methanol (−20°C) after cytospin. Cells were then washed in PBS and incubated with primary LC3B antibody (Cat. No. 3686; Cell Signaling Technology) for 1 h at room temperature. Then, cells were washed twice in PBS-Tween and once with PBS, followed by incubation with the secondary antibody (FITC-conjugated antirabbit; Cat. No. 111-096-045; Jackson ImmunoResearch) for 1 hour at room temperature. Fluorescence-labeled cells were analyzed using a confocal laser scanning microscope, and quantification of LC3B dots was performed using ImageJ.

### 4.6. Autophagy and Apoptosis Assays

Autophagy was assessed by LC3 lipidation and GFP-LC3 redistribution. For GFP-LC3 dot formation, stable GFP-LC3-expressing AML cells were cytospun, fixed with 4% paraformaldehyde for 20 min at RT, washed with PBS, and covered with fluorescent mounting solution (Dako) prior to analysis by confocal microscopy (LSM510, Carl Zeiss). To quantify GFP-LC3 dots, at least 100 cells per slide in three independent experiments were assessed, that is, the percentage of GFP-LC3-positive cells with punctuate staining; and the number of discrete puncta per cell was counted.

Tandem mCherry-EGFP-LC3B-expressing cells were treated for 2 days with 1 *μ*M ATRA. Data were acquired on a FACS LSR-II (BD) using BD FACSDiva software and analyzed with FlowJo software. A gate was used based on parental cells to estimate the percentage of high autophagic activity as previously described [49].

For annexin V staining, 0.5 × 10^6^ cells were washed with cold PBS/5% BSA, resuspended in 70 *μ*l binding buffer, and labeled with phycoerythrin- (PE-) labeled antibody against annexin V according to the manufacturer's protocol (BioVision). Caspase 3/7 activation was measured using Caspase-Glo™ 3/7 Assay according to the manufacturer's protocol (Promega Corporation, Madison, USA).

### 4.7. Statistical Analysis and Bioinformatics

Each value reported represents the mean ± SD of at least three independent experiments. *N*-fold changes were calculated using the −ΔΔCt method of relative quantification. Nonparametric Mann–Whitney *U* tests were applied to compare the difference between two groups using the program Prism software (GraphPad). *p* values < 0.05 were considered to be statistically significant. Promoter and gene sequences were retrieved from the online databases http://www.ncbi.nlm.nih.gov and http://www.ensembl.org/index.html. Putative PU.1 transcription factor binding sites were predicted by using MatInspector 8.0 software (http://www.genomatix.de).

### 4.8. Chromatin Immunoprecipitation Assay (ChIP)

ChIP was performed using the ChIP-IT Express Chromatin Immunoprecipitation Kit (Active Motif) according to the manufacturer's recommendation. Chromatin from 15 × 10^6^ NB4 cells was fragmented to an average size of 500 bp using the provided enzymatic shearing cocktail. For immunoprecipitation, we used anti-PU.1 (sc-352; Santa Cruz Biotechnology) antibody. Immunoprecipitations with IgG (PP64B; Upstate) or an anti-acetyl-histone H3 antibody (Stratagene) were used as negative and positive controls, respectively. Genomic regions containing putative PU.1 binding sites were amplified by PCR using the following primers: *ATG3* site A; forward 5′-3′: AGCATCAATCCACTCAGCATTC and reverse 5′-3′: CTGGATGGCAGTGGAAAAGAC; *ATG3* site B; forward 5′-3′: TCAGGGGTAAACTTGGAGCG and reverse 5′-3′: TTGGGATCGCAGTCACAACT; *ATG4D* site A; forward 5′-3′: CTGGAGCACTTCATTCATCCCT and reverse 5′-3′:TGAGACTGACTGCGCACC; *ATG4D* site B; forward 5′-3′: CGTTTTTGCCCCTCTCTGTA and reverse 5′-3′: CGGCTTTTAACCACCCAACC; and *ATG5*; forward 5′-3′: CAGCGTTGCCGGTTGTATTC and reverse 5′-3′: CTCCAGGCAACTACTCACCC. In addition, an unrelated sequence in the GAPDH gene was used as a negative control.

## Supplementary Materials

Supplementary 1 Supplementary Figure 1: attenuated neutrophil differentiation upon pharmacological inhibition of autophagy is not due to increased apoptosis. (a) 3-MA or CQ-mediated inhibition of autophagy impairs ATRA differentiation of HL60 cells. Cells were treated for 4 days with 1 *μ*M ATRA alone or in combination with 5 mM 3-MA or with 25 *μ*M CQ. CD11b was measured by flow cytometry as marker of neutrophil differentiation. In addition, apoptosis was blocked in the same setting using the pan-caspase inhibitor z-VAD-fmk. CD11b median fluorescence intensity (MFIs) data are mean ± s.e.m.; *n* = 1 × 104. (b) 3-MA- and CQ-mediated inhibition of autophagy resulted in increased apoptosis in ATRA-treated HL60 cells. Apoptosis was determined by annexin V staining and caspase 3/7 activity. Cells treated as in (a). Data represent the mean ± s.e.m. of three independent experiments. Mann–Whitney *U* test, ^∗^ *p* < 0.05.

Supplementary 2 Supplementary Figure 2: ATG gene expression in AML patient subtypes. ATG gene mRNA expression levels during autophagy initiation (a), nucleation (b), ATG12 (c), and LC3 (d) conjugation phases were quantified by qPCR. Analysis as in Figure 2. Mann–Whitney *U* test, ∗*p* < 0.05, ^∗∗^ *p* < 0.01, ^∗∗∗^ *p* < 0.001.

Supplementary 3 Supplementary Figure 3: ATRA-induced autophagy and neutrophil differentiation of AML cells is Beclin1-independent. (a) Minor Beclin1 protein induction during ATRA differentiation of NB4, HL60, and HT93 AML cells. Western blots of Beclin1 and GAPDH are shown. (b) Inhibition of Beclin1 did not abrogate ATRA-induced autophagy as measured by LC3B lipidation. Cells stably expressing a scramble control shRNA (shCtrl) or shRNAs targeting Beclin1 (shBeclin1) were treated with ATRA for 4 days. GAPDH was used as a loading control. (c) Inhibition of Beclin1 does not prevent ATRA-induced GFP-LC3 puncta formation. Left panels: HL60 and NB4 GFP-LC3 cells stably expressing a scramble control shRNA (shCtrl) or shRNAs targeting Beclin1 (shBeclin1) were treated with ATRA for 4 days. The percentage of GFP-LC3 puncta-positive cells and average numbers of puncta were quantified by confocal microscopy. Counts are mean ± s.e.m.; *n* = 100; three independent experiments; n.s.: not significant. Right panels: starvation- and arsenic trioxide- (As_2_O_3_) induced autophagy are inhibited by knocking down Beclin1. Cells were starved or treated with As_2_O_3_, and autophagic activity was assessed by confocal microscopy. Counts are mean ± s.e.m.; *n* = 100; three independent experiments. (d) Knocking down Beclin1 does not impair neutrophil differentiation of AML cells as determined by CD11b expression. Data are mean ± s.e.m.; *n* = 1 × 104. Mann–Whitney *U* test, ^∗^ *p* < 0.05. n.s.: not significant.
