# Supplementary figures and images for: Low Autophagy (ATG) Gene Expression Is Associated with an Immature AML Blast Cell Phenotype and Can Be Restored during AML Differentiation Therapy
Source: Oxid Med Cell Longev. 2018 Mar 18;2018:1482795. doi: 10.1155/2018/1482795 (PMC5878891; doi:10.1155/2018/1482795)

## Slide 1
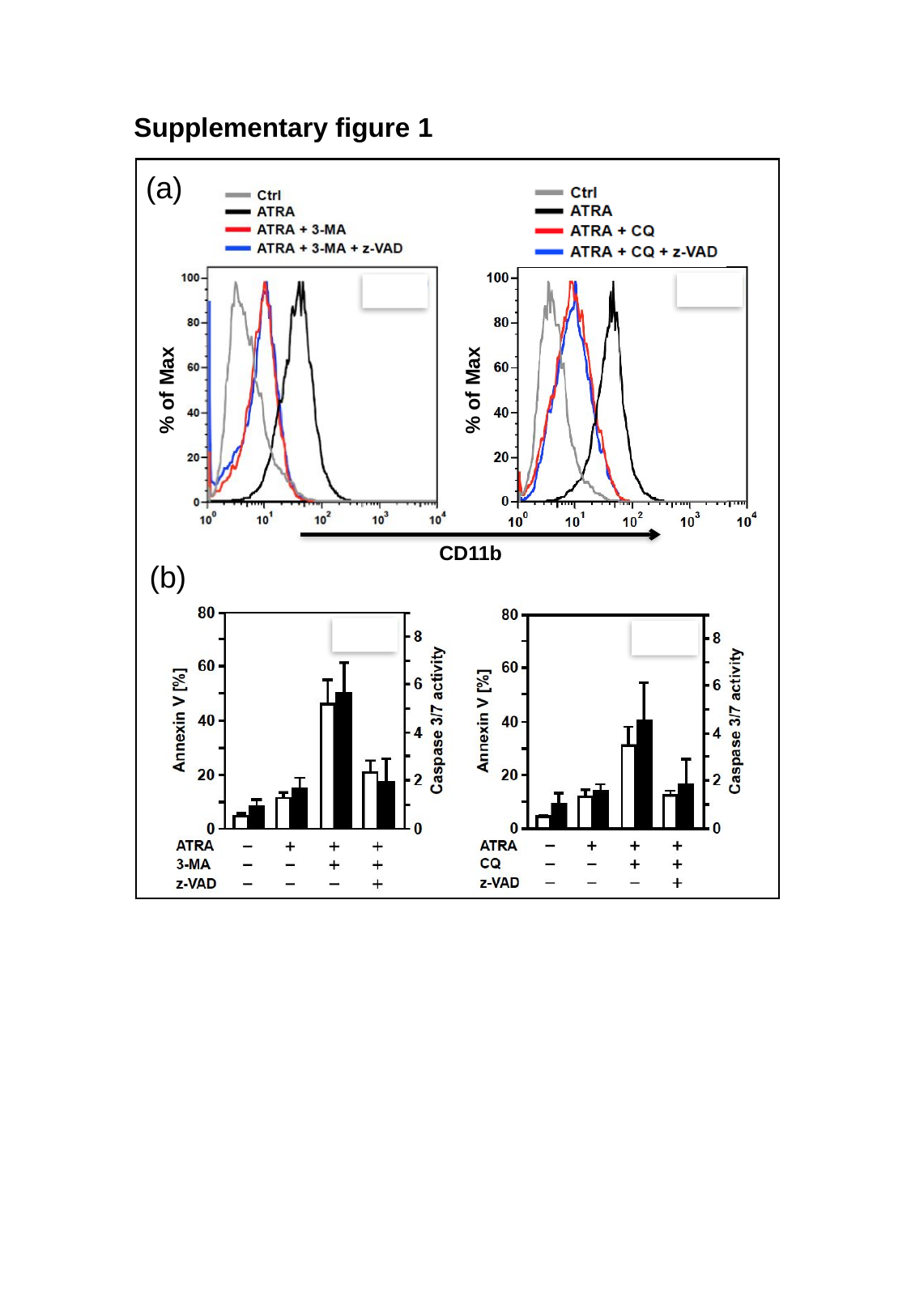

Supplementary figure 1
(a)
% of Max
% of Max
CD11b
(b)

Supplement: Supplementary 1 — Supplementary Figure 1: attenuated neutrophil differentiation upon pharmacological inhibition of autophagy is not due to increased apoptosis. (a) 3-MA or CQ-mediated inhibition of autophagy impairs ATRA differentiation of HL60 cells. Cells were treated for 4 days with 1 μM ATRA alone or in combination with 5 mM 3-MA or with 25 μM CQ. CD11b was measured by flow cytometry as marker of neutrophil differentiation. In addition, apoptosis was blocked in the same setting using the pan-caspase inhibitor z-VAD-fmk. CD11b median fluorescence intensity (MFIs) data are mean ± s.e.m.; n = 1 × 10^4^. (b) 3-MA- and CQ-mediated inhibition of autophagy resulted in increased apoptosis in ATRA-treated HL60 cells. Apoptosis was determined by annexin V staining and caspase 3/7 activity. Cells treated as in (a). Data represent the mean ± s.e.m. of three independent experiments. Mann–Whitney U test, ∗ p < 0.05. [file 1482795.f1.pptx]

## Slide 1
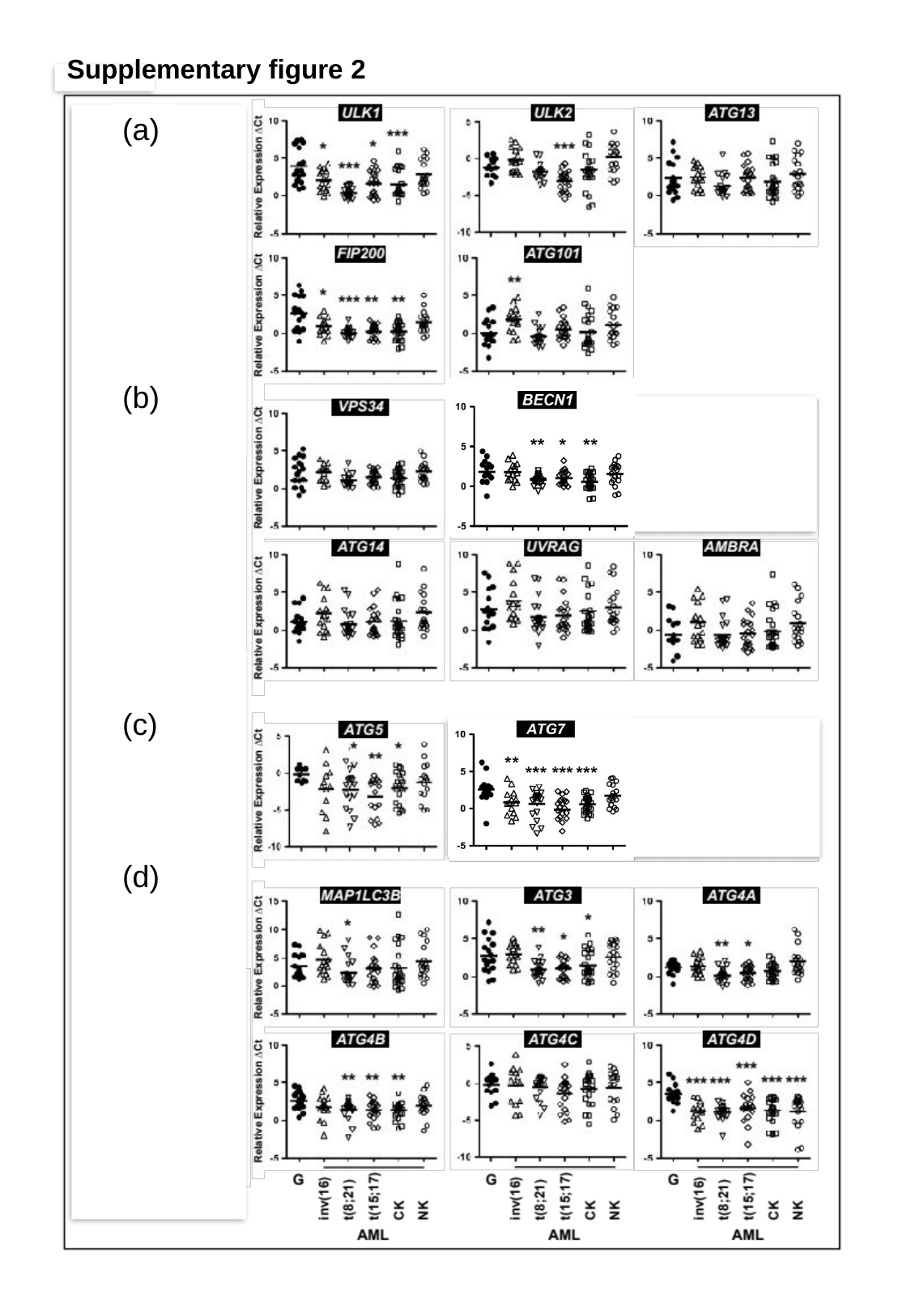

Supplementary figure 2
(a)
(b)
(c)
(d)

Supplement: Supplementary 2 — Supplementary Figure 2: ATG gene expression in AML patient subtypes. ATG gene mRNA expression levels during autophagy initiation (a), nucleation (b), ATG12 (c), and LC3 (d) conjugation phases were quantified by qPCR. Analysis as in Figure 2. Mann–Whitney U test, ∗p < 0.05, ∗∗ p < 0.01, ∗∗∗ p < 0.001. [file 1482795.f2.pptx]

## Slide 1
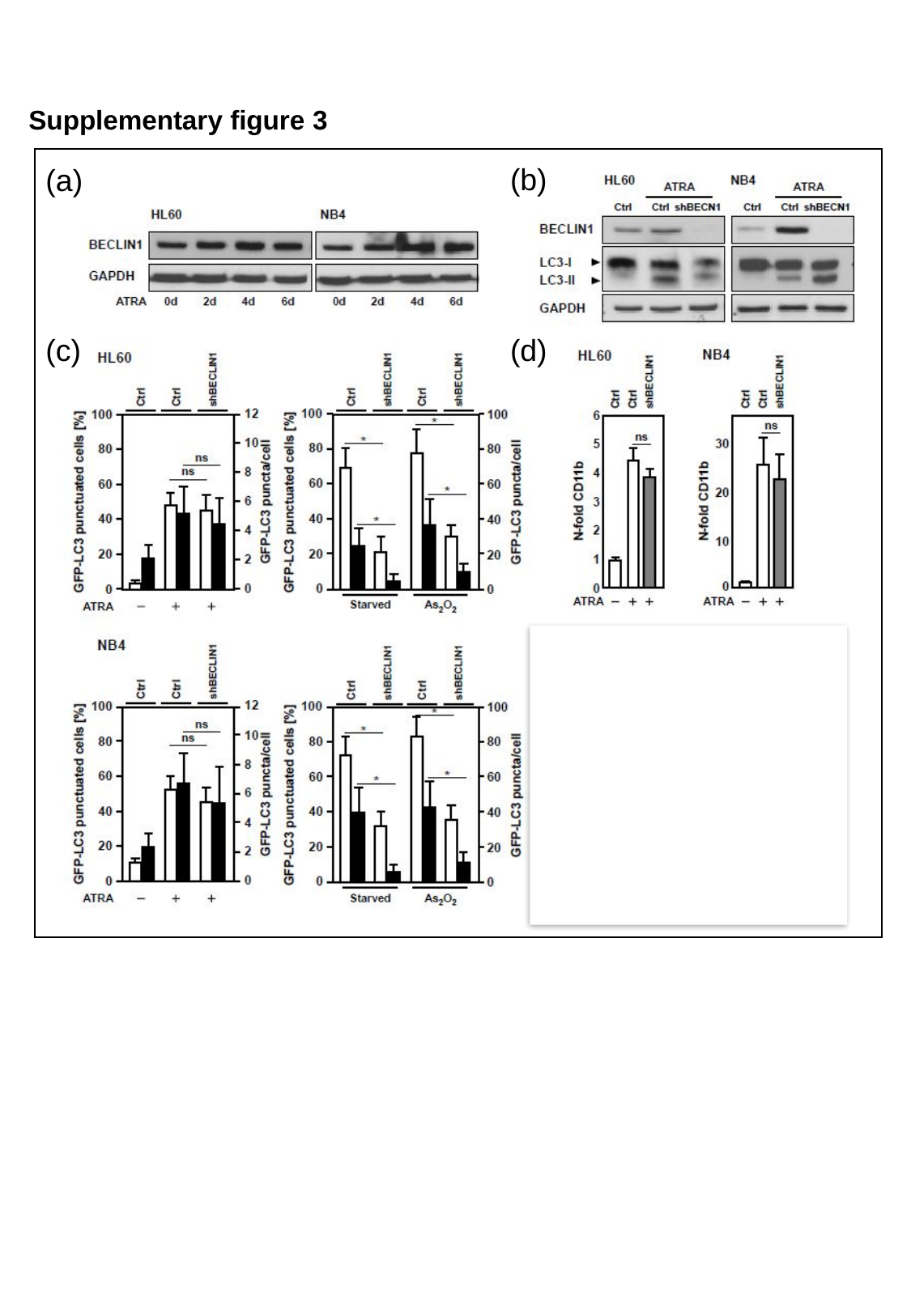

Supplementary figure 3
(b)
(a)
(c)
(d)

Supplement: Supplementary 3 — Supplementary Figure 3: ATRA-induced autophagy and neutrophil differentiation of AML cells is Beclin1-independent. (a) Minor Beclin1 protein induction during ATRA differentiation of NB4, HL60, and HT93 AML cells. Western blots of Beclin1 and GAPDH are shown. (b) Inhibition of Beclin1 did not abrogate ATRA-induced autophagy as measured by LC3B lipidation. Cells stably expressing a scramble control shRNA (shCtrl) or shRNAs targeting Beclin1 (shBeclin1) were treated with ATRA for 4 days. GAPDH was used as a loading control. (c) Inhibition of Beclin1 does not prevent ATRA-induced GFP-LC3 puncta formation. Left panels: HL60 and NB4 GFP-LC3 cells stably expressing a scramble control shRNA (shCtrl) or shRNAs targeting Beclin1 (shBeclin1) were treated with ATRA for 4 days. The percentage of GFP-LC3 puncta-positive cells and average numbers of puncta were quantified by confocal microscopy. Counts are mean ± s.e.m.; n = 100; three independent experiments; n.s.: not significant. Right panels: starvation- and arsenic trioxide- (As2O3) induced autophagy are inhibited by knocking down Beclin1. Cells were starved or treated with As2O3, and autophagic activity was assessed by confocal microscopy. Counts are mean ± s.e.m.; n = 100; three independent experiments. (d) Knocking down Beclin1 does not impair neutrophil differentiation of AML cells as determined by CD11b expression. Data are mean ± s.e.m.; n = 1 × 10^4^. Mann–Whitney U test, ∗ p < 0.05. n.s.: not significant. [file 1482795.f3.pptx]
